# The reversal of antineoplastic drug resistance in cancer cells by β-elemene

**DOI:** 10.1186/s40880-015-0048-0

**Published:** 2015-09-14

**Authors:** Guan-Nan Zhang, Charles R. Ashby, Yun-Kai Zhang, Zhe-Sheng Chen, Huiqin Guo

**Affiliations:** Department of Pharmaceutical Sciences, College of Pharmacy and Health Sciences, St. John’s University, Queens, NY 11439 USA; Department of Thoracic Surgery, Peking Union Medical College Hospital, Beijing, 100730 P. R. China

**Keywords:** *Rhizoma zedoariae*, β-Elemene, Multidrug resistance

## Abstract

Multidrug resistance (MDR), defined as the resistance of cancer cells to compounds with diverse structures and mechanisms of actions, significantly limits the efficacy of antitumor drugs. A major mechanism that mediates MDR in cancer is the overexpression of adenosine triphosphate (ATP)-binding cassette transporters. These transporters bind to their respective substrates and catalyze their efflux from cancer cells, thereby lowering the intracellular concentrations of the substrates and thus attenuating or even abolishing their efficacy. In addition, cancer cells can become resistant to drugs via mechanisms that attenuate apoptosis and cell cycle arrest such as alterations in the p53, check point kinase, nuclear factor kappa B, and the p38 mitogen-activated protein kinase pathway. In this review, we discuss the mechanisms by which β-elemene, a compound extracted from *Rhizoma zedoariae* that has clinical antitumor efficacy, overcomes drug resistance in cancer.

## Background

**Fig. 1:**
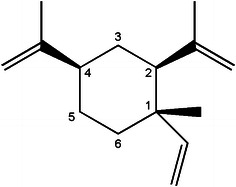
Structure of β-elemene

*Curcuma* belongs to the *Zingiberaceae* family and is a medicinal plant that distributes worldwide. The plants of this genus are mainly grown in southeastern Asia, Brazil, and Australia [1]. Recently, approximately 20 *Curcuma* species have been discovered in China [2]. The Chinese Pharmacopoeia indicates that *Rhizoma zedoariae* is the dry rhizome derived from *Curcumawenyujin* [1], *Curcumaphaeocaulis* [2], and *Curcumakwangsiensis* [3]. *Rhizoma zedoariae* has been used as an anti-microbial, anti-inflammatory, anti-proliferative, and antitumor drug [3–7]. β-elemene [(1S,2S,4R)-2,4-diisopropenyl-1-methyl-1-vinylcyclohexane], a naturally occurring compound isolated from *Rhizoma zedoariae*, is approved for use in Chinese medicine to treat a variety of cancers, including leukemia and brain, breast, prostate, ovarian, cervical, and lung cancers [8–14]. The structure of β-elemene is shown in Fig. 1. β-elemene does not produce significant or problematic toxicity and is well tolerated by patients [12]. It has been postulated that the anticancer effect of β-elemene is due to the induction of apoptosis and cell cycle arrest [8, 14].

Currently used antitumor drugs can produce their therapeutic action via a number of distinct mechanisms. For example, antitumor drugs can (1) damage DNA or inhibit DNA replication, (2) inhibit DNA and RNA synthesis, (3) interfere with RNA transcription, (4) inhibit protein synthesis, (5) interfere with hormone homeostasis, and (6) disrupt cellular microtubules via stabilization or destabilization [15]. However, it is well established that cancer cells can become resistant to many antitumor drugs; this phenomenon is known as multidrug resistance (MDR) [16]. MDR occurs when cancer cells become resistant to a variety of drugs that have distinct structures and mechanisms of action [17]. A number of mechanisms have been reported to produce MDR, including altered activity of specific enzyme systems such as glutathione S-transferase (GST) and topoisomerase, which can attenuate the efficacy of anticancer drugs [18, 19]. In addition, alterations in the levels of proteins that control apoptosis can occur; such alterations decrease the efficacy of anticancer drugs by inducing apoptosis [20]. A well-documented mechanism that produces or elicits MDR is the overexpression of adenosine triphosphate (ATP)-binding cassette (ABC) transporters, such as the ABCB1 (P-glycoprotein, P-gp/MDR1), ABCCs [multidrug resistance-associated proteins (MRPs)], and ABCG2 transporters (BCRP/MXR/ABCP) [21]. These transporters use energy obtained from the hydrolysis of ATP to remove or efflux compounds from cancer cells, thereby significantly lowering their intracellular concentrations and attenuating their efficacy [22]. The ABC transporter P-gp is expressed by cancer cells derived from epithelial cells of the colon, liver, adrenal gland, and pancreas and has been reported to produce resistance to a broad spectrum of anticancer drugs, including anthracyclines, vinca alkaloids, etoposide, and taxanes [23–26]. In addition, the overexpression of other ABC transporters, such as ABCCs and ABCG2, can also produce MDR in cancer cells [21, 27–29]. Numerous studies indicate that the blockade of the efflux function of specific ABC transporters by various compounds can resensitize resistant cancer cells to specific antitumor drugs by increasing the intracellular concentration of these transporters. However, this approach has been hampered because inhibitors of the efflux activity of ABC transporters have produced severe adverse effects and toxic drug–drug interactions [30].

One approach that has been used to overcome MDR in cancer cells is to find or synthesize compounds that can block the efflux action of the ABC transporters without producing significant toxic effects [31]. Over the last four decades, three generations of ABC transporter inhibitors have been developed in an attempt to overcome MDR in cancer cells. One of the first-generation drugs, verapamil, a calcium channel blocker, was the first drug shown to inhibit the efflux function of P-gp [32]. However, clinical data indicated that verapamil produced significant toxic effects at concentrations required to overcome P-gp-mediated resistance [33]. In addition, verapamil and other first-generation compounds were shown to inhibit the activities of various cytochrome P450 (CYP450) enzymes, thereby increasing the likelihood of adverse and toxic drug–drug interactions [34–36]. To overcome the aforementioned limitations of first-generation compounds, second-generation compounds were developed [37, 38]. Second-generation P-gp modulators included PSC833 and biricodar (VX-710). Compared with first-generation compounds, these P-gp inhibitors were reported to be more potent in reversing MDR and less toxic [37, 39]. Among second-generation P-gp modulators, PSC833 was the most well characterized, and it was used in clinical trials in combination with doxorubicin [DOX, also known as adriamycin (ADM)], vincristine, vinblastine, paclitaxel (TAX), or mitoxantrone to treat MDR cancer [37, 39]. PSC833 alone did not produce significant adverse effects, but it increased the likelihood of toxic effects when used with antitumor drugs. For example, a significant pharmacokinetic interaction was observed when PSC833 was administered in combination with DOX and TAX [40, 41], thus requiring a reduction in the doses of the anticancer drugs. It was subsequently shown that second-generation P-gp inhibitors significantly decreased the excretion of various drugs, thereby increasing the incidence of significant adverse effects [41]. In addition, to overcome the problematic drug–drug interactions, the doses of these drugs had to be decreased, which attenuated their efficacy.

Third-generation P-gp inhibitors such as tariquidar (XR9576), laniquidar (R101933), and LY335979, were developed to overcome the limitations associated with second-generation inhibitors [42–45]. Although third-generation P-gp inhibitors were significantly efficacious in vitro, these compounds are not used clinically because in vivo and preclinical studies indicated a lack of efficacy and/or significant adverse effects [46].

**Fig. 2:**
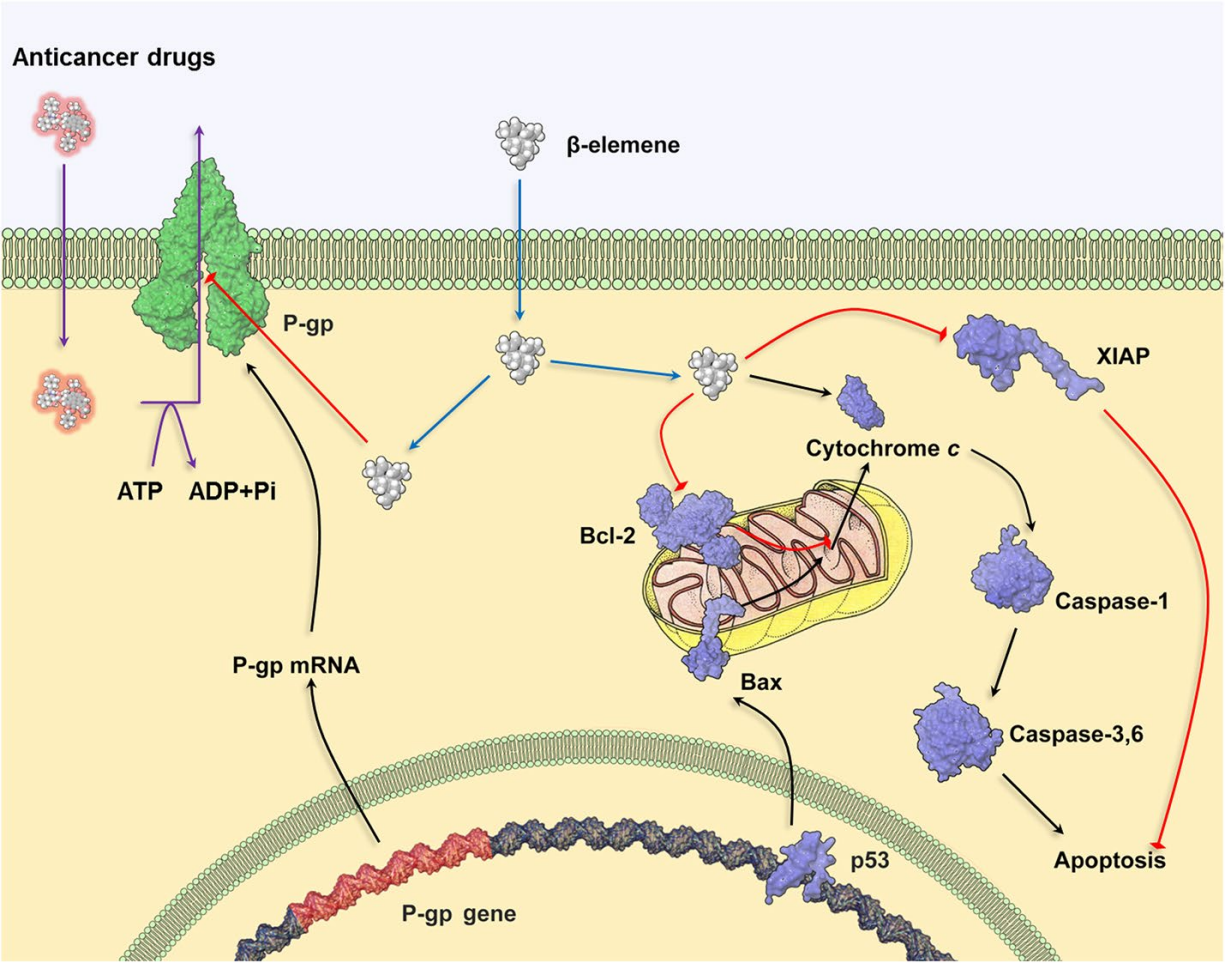
A schematic model of the mechanisms for β-elemene-induced apoptosis and augmentation of the efficacy of anticancer drugs. β-elemene may enhance the therapeutic effect of anticancer drugs by blocking the substrate efflux function (*purple arrows*) of the multidrug-resistant transporter P-glycoprotein (P-gp). In addition, it has been proposed that β-elemene may affect multiple pathways to produce apoptosis in tumor cells. *Black arrows* indicate stimulatory modifications, whereas *red arrows* indicate inhibitory modifications. *XIAP* X-linked inhibitor of apoptosis protein, *ATP* adenosine triphosphate, *ADP* adenosine diphosphate

It has been well-established that certain compounds extracted from various natural sources have antitumor efficacy [47, 48]. However, relatively few studies have reported the effects of naturally derived compounds on the activities and functions of ABC transporters. Given the important role of ABC transporters in mediating MDR, we recently reported the in vitro effect of the compound β-elemene, extracted from the plant *Rhizoma zedoariae*, on MDR in cancer cells [49]. In addition, this compound may overcome MDR via other mechanisms [50]. Therefore, in this review, we discuss the mechanisms by which β-elemene surmounts MDR. Figure 2 illustrates the mechanisms by which β-elemene mediates the reversal of MDR in cancer cells.

### Antitumor efficacy of β-elemene

β-Elemene has been purported to inhibit the proliferation of cancer cells by inducing apoptotic cell death and cell cycle arrest [8, 51, 52]. The dysfunction or blockade of apoptosis has been proposed to play a role in abnormal cell proliferation, thus initiating the carcinogenic processes involved in tumor proliferation, angiogenesis, and metastasis [53]. Apoptosis can be initiated by the activation of the intrinsic and extrinsic pathways. The activation of the intrinsic pathway disrupts the balance between pro-survival proteins (e.g., Bcl-2 and Bcl-xL) and pro-apoptotic proteins of the Bcl-2 family (e.g., Bax and Bak), which triggers the release of cytochrome *c* from the mitochondrial outer membrane [54]. In contrast, the extrinsic pathway is activated by the binding of specific molecules to death receptors such as FAS receptor (FasR), tumor necrosis factor receptor 1 (TNFR1), death receptor 3 (DR3), and death receptor 4/death receptor 5 (DR4/DR5) [55]. Numerous studies indicate that apoptosis is an important therapeutic target for cancer treatment [56]. There are studies indicating that β-elemene affects the apoptotic process in cancer cells. For example, β-elemene significantly inhibits the growth and proliferation of various types of T24 bladder cancer cell lines by decreasing the expression of the anti-apoptotic proteins Mta-1, Survivin, and Bcl-xL [57]. In addition, β-elemene significantly inhibits the proliferation of lung and prostate cancer cells by increasing the release of cytochrome *c* and the activation of caspases-3, -7, and -9 and of poly(ADP-ribose) polymerase (PARP) and by decreasing the expression of Bcl-2 [58].

There are data suggesting that β-elemene triggers cell cycle arrest by activating the p38 mitogen-activated protein kinase (MAPK) pathway. For example, in C6 glioblastoma cells, β-elemene significantly increases the fraction of C6 cells at the G_0_/G_1_ phase [48]. The cell cycle-arresting action of β-elemene was associated with an increase in the phosphorylation of p38 MAPK, whereas this effect was reversed by the inhibition of p38 MAPK [52]. Furthermore, in non-small cell lung cancer (NSCLC) and epithelial cell lines, β-elemene significantly arrested the cell cycle at the G_2_-M phase by decreasing the expression of Cyclin B1 and phospho-Cdc2 (Thr-161) and by increasing the expression of P27 (kip) and phospho-Cdc (Tyr-15) [11]. Recently, Zhao et al. [59] reported that β-elemene can significantly inhibit the proliferation of NSCLC cells by inhibiting extracellular signal-regulated kinases (ERK1/2) and the adenosine monophosphate-activated protein kinase α (AMPKα)-mediated transcription factor Sp1 and then by decreasing the protein expression of DNA (cytosine-5)-methyltransferase 1 (DNMT1). In addition, the action of β-elemene on the proliferation of NSCLC cells was reversed by the overexpression of DNMT1, and the inhibition of Akt signaling and DNMT1 expression by metformin can potentiate the effects of β-elemene [59]. In A2780/CP ovarian carcinoma cells (which are resistant to cisplatin), β-elemene induces cell cycle arrest at the G_2_/M phase by decreasing the expression of B1 and Cdc2 and by increasing the expression of p53, p27, and growth arrest and DNA-damage-inducible protein (GADD45) [8].

It is well documented that radiation therapy plays an important role in the treatment of radiation-sensitive tumors [60]. The overexpression of peroxiredoxin 1 (Prx-1), a critical regulator of redox in cancer cells, has been reported to abrogate the response of cancer cells to radiation therapy [60]. Thus, reducing the expression of Prx-1 is a promising way to resensitize tumor cells to radiation therapy. In this study, Li et al. [60] demonstrated that 45 mg/kg β-elemene restores the sensitivity of A549 tumor cells to radiation. Reverse transcription-polymerase chain reaction (RT-PCR) and Western blotting assays indicated that the potentiation of radiation therapy by β-elemene was mediated by the down-regulation of Prx-1.

### Reversal of MDR by β-elemene

#### Reversal of ABC transporter-mediated MDR by β-elemene

As mentioned above, one of the well-documented mechanisms responsible for MDR is the overexpression of P-gp. Therefore, our laboratory conducted a study to examine the in vitro effect of β-elemene on P-gp-induced MDR in drug-resistant and non-drug-resistant parental cell lines [49]. We determined the effect of β-elemene on the cytotoxic effects of the antitumor drugs colchicine, vinblastine, and TAX (P-gp substrates) in parenteral KB-3-1 cells and in KB-C2 cells overexpressing P-gp. β-elemene (100 μmol/L) significantly increased the efficacy (i.e., the cytotoxicity) of P-gp substrate drugs in KB-C2 cells but not in parenteral KB-3-1 cells. For example, the responses to TAX, vinblastine, and colchicine in KB-C2 cells were 648-, 11.7-, and 720-fold lower, respectively, than those in parental KB-3-1 cells. β-elemene (100 μmol/L) significantly lowered the magnitude of resistance to TAX, vinblastine, and colchicine (by 4.6-, 5.4-, and 1.1-fold, respectively) in KB-C2 cells. The results of accumulation and efflux assays indicated that β-elemene potentiated the cytotoxic action of the antitumor drugs that were P-gp substrates by blocking their efflux via P-gp, thereby increasing their intracellular concentrations (by 3.6-fold) in KB-C2 cells overexpressing P-gp. It is possible that β-elemene could potentiate the actions of antitumor drugs by decreasing the expression of P-gp protein. However, the incubation of KB-C2 with β-elemene for 24, 48, or 72 h did not significantly alter the expression of P-gp. Overall, our results suggested that β-elemene potentiated the actions of P-gp substrate drugs by inhibiting the efflux function of P-gp. Our study did not rule out the possibility that β-elemene could produce its potentiating action by decreasing the insertion of the P-gp transporter into the cell membrane.

Similar to our findings, Xu et al. [61] reported that β-elemene (30 μmol/L) significantly potentiated (6.38-fold) the action of DOX in DOX-resistant MCF-7 cells. Furthermore, it was shown that β-elemene (30 μmol/L) significantly increased the intracellular accumulation of DOX and the compound Rh123, which is a substrate of P-gp. Interestingly, Xu et al. [61] showed that β-elemene (30 μmol/L) significantly decreased P-gp protein expression, suggesting that the action of β-elemene is due in part to this mechanism. This finding was in contrast to our results indicating that β-elemene did not significantly alter the expression of P-gp. The exact explanation for this discrepancy is unknown, but it may be due to differences in the cell lines used (MCF-7/DOX versus KB-C2 and HEK293/ABCB1) [61].

β-Elemene significantly increases the suppressive effect of DOX and docetaxel (Doc) on the growth and proliferation of the resistant MCF-7/Adr and MCF-7/Doc breast cancer cell lines [7]. β-elemene may attenuate MDR by influencing MDR-related microRNA expression and subsequently regulating the target genes phosphatase and tensin homolog (*PTEN*) and *P*-*gp*, which are responsible for the proliferation of resistant breast cancer cells [7].

#### β-Elemene reverses MDR by promoting apoptosis of resistant cancer cells

It is known that mechanisms that inhibit or attenuate the induction of cancer cell apoptosis produce resistance to certain antitumor drugs [20]. The induction of apoptosis by antitumor drugs has been shown to trigger a significant generation of reactive oxygen species (ROS) and disruption of mitochondrial membrane potential [62]. Numerous studies suggest that apoptosis involves the complex interaction and regulation of many genes and proteins [56]. The suppressor gene *p53* is a key mediator of apoptosis-induced cellular transformation [63, 64]. Following DNA damage by certain antitumor drugs, *p53* is activated, resulting in G_1_ phase arrest and, subsequently, apoptosis [56, 65]. Thus, the loss of *p53* or inactivation of the p53 pathway could contribute to drug resistance [63, 66]. In addition, proteins whose expression is regulated by *p53*, such as B cell lymphoma-2 (Bcl-2) and Bax, are also involved in mediating resistance to antitumor drugs [67]. Indeed, proteins categorized as anti-apoptotic Bcl-2 members are transcriptionally up-regulated in cancer cells and are associated with resistance to the antitumor drugs DOX, TAX, cisplatin, mitoxantrone, and etoposide. Thus, the down-regulation (as well as inactivation) of anti-apoptotic Bcl-2 proteins can augment apoptosis, thereby increasing the efficacy of antitumor drugs in resistant cancer cells. For example, it has been postulated that the reversal mechanism of β-elemene (6 μg/mL) in the ADM-resistant human breast cancer cell line MCF-7/ADM may result from a decrease in the expression of Bcl-2 [68]. In the parental ovarian cancer cell line A2780 and the cisplatin-resistant cell line A2780/CP70, β-elemene (0.6 μmol/L**)** significantly enhanced cisplatin cytotoxicity in the drug-resistant cell lines (by 60-fold) [69]. These results suggested that β-elemene acts by abrogating the expression of the excision pathway that repairs protein cross-complementation group 1 (ERCC1) protein. In addition, the level of the X-linked inhibitor of apoptosis protein (XIAP) is significantly decreased by β-elemene (0.6 μmol/L) [69, 70]. In ovarian carcinoma cells resistant to cisplatin, the combination of β-elemene and cisplatin can stimulate the activity of caspases-3, -8, and -9 as well as the cleavage of caspase-9, whereas the expression of Bcl-2 and Bcl-xL was decreased [70]. Thus, based on the aforementioned results, β-elemene restores the sensitivity of resistant ovarian cancer cells to cisplatin by attenuating DNA repair activity and promoting apoptosis. Furthermore, β-elemene significantly augments the antitumor activity of cisplatin in human bladder cancer cells by inducing cell apoptosis via a caspase-dependent mechanism [69, 70]. Wang et al. [11] reported that β-elemene significantly augments cisplatin-induced inhibition of the growth of a NSCLC cell line by inducing cell cycle arrest. Similarly, β-elemene significantly increased the suppressive effect of cisplatin on the growth and proliferation of NSCLC H460 and A549 cell lines [14]. It was postulated that the potentiation of the efficacy of cisplatin by β-elemene was due to inducing cell cycle arrest in NSCLC cells at the G_2_/M phase by increasing checkpoint kinase 2 (CHK2) expression and reducing Cdc2 activity [71]. In addition, the combination of β-elemene and cisplatin significantly decreased the protein levels of Cyclin B1 and Cdc25C and increased the levels of P21, P27, and GADD45 in cancer cells [71]. Furthermore, a meta-analysis of clinical data suggested that the combination of β-elemene and cisplatin was more efficacious than cisplatin alone in treating NSCLC and significantly improved the quality of life of the patients [22].

Endocrine therapy plays a critical role in the treatment of estrogen receptor (ER)-positive breast cancer, and the lack of ER expression is associated with a decrease in the efficacy of endocrine-based therapy [72]. In MCF7/TAX cell lines, which do not express ER-α, 10 μg/mL of β-elemene restored the sensitivity of MCF7/TAM cell lines to TAX. RT-PCR and Western blotting assays indicated that the reversal of TAX resistance by β-elemene was mediated by up-regulating the expression of *ER*-*α* mRNA via the MAPK pathway [73].

The protein nuclear factor-κB (NF-κB) is associated with the development of chemo-resistance in various cancer cells by inducing the overexpression of anti-apoptotic proteins [74]. Thus, inhibition of NF-κB-mediated responses may be a promising strategy for potentiating the response to antitumor drugs. The incubation of ADM-resistant cells (SGC7901/ADM gastric cancer cells) with β-elemene significantly decreased the expression of NF-κB [75]. In addition, β-elemene significantly increased the apoptotic rate of SGC7901/ADM cells by inhibiting or attenuating NF-κB activity [75].

#### β-Elemene reverses MDR by reducing the stemness of cancer cells

Glioblastoma stem-like cells (GSCs) play an important role in tumor development, recurrence, and chemo-resistance [76]. It has been postulated that impairing stemness and enhancing differentiation could decrease GSC-associated chemo-resistance [76]. Fu et al. [75] reported that β-elemene significantly impaired the stemness of GSC spheres, dispersed GSC spheres, and reduced the proliferation of GSCs in vitro and in vivo. In addition, they also found that β-elemene in GSC spheres and xenografts can significantly decrease the expression of CD133 and ABCG2 and increase the expression of glial fibrillary acidic protein (GFAP). Furthermore, β-elemene can also restore the sensitivity of GSCs to temozolomide [76]. The β-elemene-mediated potentiation of the efficacy of temozolomide on glioblastoma cells results from activation of the glia maturation factor β (GMFβ)/MAPK 3/6/p38 pathway [77]. In addition, it has been reported that the antitumor effect of β-elemene was also mediated by GMFβ-dependent inactivation of the ERK1/2-Bcl-2/survivin pathway, which suggests that β-elemene is a promising chemosensitizer for temozolomide against glioblastoma tumors [77].

In addition, the combination of β-elemene and TAX, compared with TAX alone, significantly inhibited the proliferation of MB-468 cells by decreasing the expression of Cyclin B1 and increasing the expression of P27 (kip1) [78]. Furthermore, the combination of β-elemene (20 or 50 μg/mL) and etoposide phosphate (VP-16, 15 μg/mL) increased the likelihood of apoptosis compared with VP-16 alone (16.57 or 21.98 vs. 6.25%) [79]. The potentiation of the efficacy of VP-16 by β-elemene may be mediated by the increase of Bax, p53, and p21 and the suppression of Cyclin D1 [79].

## Conclusions

β-Elemene plays an important role in overcoming MDR via multiple mechanisms. It is possible that the reversal action of β-elemene could result from (1) a blockade of the efflux function of P-gp, (2) a decrease in the protein levels of P-gp, (3) an induction of apoptosis or cell cycle arrest, or (4) a decrease in NF-κB signaling activity. Finally, it may be of interest to determine if β-elemene alters the expression and/or efflux activity of other ABC transporters. Currently, the in vitro and in vivo data obtained for β-elemene suggest that it may be useful in treating certain MDR cancers. However, controlled clinical trials will be required to determine if β-elemene will significantly increase the efficacy of various chemotherapeutic drugs in reversing MDR in cancer patients.
